# Author Correction: Perception, performance, and detectability of conversational artificial intelligence across 32 university courses

**DOI:** 10.1038/s41598-023-43998-8

**Published:** 2023-10-10

**Authors:** Hazem Ibrahim, Fengyuan Liu, Rohail Asim, Balaraju Battu, Sidahmed Benabderrahmane, Bashar Alhafni, Wifag Adnan, Tuka Alhanai, Bedoor AlShebli, Riyadh Baghdadi, Jocelyn J. Bélanger, Elena Beretta, Kemal Celik, Moumena Chaqfeh, Mohammed F. Daqaq, Zaynab El Bernoussi, Daryl Fougnie, Borja Garcia de Soto, Alberto Gandolfi, Andras Gyorgy, Nizar Habash, J. Andrew Harris, Aaron Kaufman, Lefteris Kirousis, Korhan Kocak, Kangsan Lee, Seungah S. Lee, Samreen Malik, Michail Maniatakos, David Melcher, Azzam Mourad, Minsu Park, Mahmoud Rasras, Alicja Reuben, Dania Zantout, Nancy W. Gleason, Kinga Makovi, Talal Rahwan, Yasir Zaki

**Affiliations:** 1 Division of Science, New York University Abu Dhabi, Abu Dhabi, UAE; 2 Division of Social Science, New York University Abu Dhabi, Abu Dhabi, UAE; 3 Division of Engineering, New York University Abu Dhabi, Abu Dhabi, UAE

Correction to: *Scientific Reports* https://doi.org/10.1038/s41598-023-38964-3, published online 24 August 2023

In the original version of this Article Dania Zantout was incorrectly affiliated with ‘Division of Social Science, New York University Abu Dhabi, Abu Dhabi, UAE’. The correct affiliation is listed below.

Division of Science, New York University Abu Dhabi, Abu Dhabi, UAE

The original Article has been corrected.

