# Supplementary material for: Perception, performance, and detectability of conversational artificial intelligence across 32 university courses
Source: Sci Rep. 2023 Aug 24;13:12187. doi: 10.1038/s41598-023-38964-3 (PMC10449897; doi:10.1038/s41598-023-38964-3)
Supplement: Supplementary file 1 — Supplementary Information. [file 41598_2023_38964_MOESM1_ESM.pdf]

Supplementary Information for

Perception, performance, and detectability of conversational artificial intelligence across 32 university courses

Hazem Ibrahim[1], Fengyuan Liu[1], Rohail Asim[1], Balaraju Battu[1], Sidahmed Benabderrahmane[1], Bashar Alhafni[1], Wifag Adnan[2], Tuka Alhanai[3], Bedoor AlShebli[2], Riyadh Baghdadi[1], Jocelyn J. Bélanger[1], Elena Beretta[1], Kemal Celik[3], Moumena Chaqfeh[1], Mohammed F. Daqaq[3], Zaynab El Bernoussi[2], Daryl Fougnie[1], Borja Garcia de Soto[3], Alberto Gandolfi[1], Andras Gyorgy[3], Nizar Habash[1], J. Andrew Harris[2], Aaron Kaufman[2], Lefteris Kirousis[1], Korhan Kocak[2], Kangsan Lee[2], Seungah S. Lee[2], Samreen Malik[2], Michail Maniatakos[3], David Melcher[1], Azzam Mourad[1], Minsu Park[2], Mahmoud Rasras[3], Alicja Reuben[2], Dania Zantout[1], Nancy W. Gleason[2], Kinga Makovi[2], Talal Rahwan[1*], Yasir Zaki[1*]

1 Division of Science, New York University Abu Dhabi, UAE
2 Division of Social Science, New York University Abu Dhabi, UAE
3 Division of Engineering, New York University Abu Dhabi, UAE
* To whom correspondence should be addressed; E-mail: {talal.rahwan, yasir.zaki}@nyu.edu.

This document is structured as follows:

- **Supplementary Tables 1 to 7** (*page 2*)
- **Supplementary Note 1** (*page 12*) Survey Questions
- **Supplementary Figures** (*page 31*)

Supplementary Tables

Supplementary Table 1: **Inter-rater reliability of standardized grades given to each submission.** Inter-rater reliability is reported as the Krippendorff′s alpha coefficient.

**Course**	**Overall inter-grader reliability**
Education and Society	0.98
Computer Systems Organization	0.97
Calculus with applications to Science and Engineering	0.94
Data Structures	0.93
Political Economy of Institutions	0.9
Applied Data Science using Python	0.89
Quantiative Synthetic Biology	0.88
Modeling and Analysis of Dynamical Systems	0.88
Project Management	0.86
Mathematics for Statistics and Calculus	0.83
Structure and Properties of Civil Engineering Materials	0.82
Intro to the Study of Society	0.79
Markets	0.76
Social Psychology	0.75
Algorithms	0.75
Data Analysis	0.73
Operating Systems	0.7
Management and Organizations	0.69
Multivariable Calculus with applications to Economics	0.67
Intro to Computer Science	0.66
Computer Networks	0.65
Intro to Public Policy	0.63
Intro to Psychology	0.63
Discrete Mathematics	0.63
Natural Language Processing	0.62
Biopsychology	0.6
Human Centered Data Science	0.54
Objected Oriented Programming	0.48
The Sociology of Entrepreneurship	0.48
Cyberwarfare	0.47
Corporate Finance	0.4
Climate/Change	0.1

Supplementary Table 2: **Comparing ChatGPT's average grades to those of university students in 32 different course.** p-values are calculated using bootstrapped Welch's t-test. Ticks highlight courses where the average grade of GPT submissions is not significantly lower than that of students. SRPP stands for Social Research and Public Policy.

**Discipline**	**Course**	**Avg. student grade**	**Avg. GPT grade**	**t-statistics**	**p-values**	**GPT ≥ Human**
Computer Science	Computer Systems Organization	9.83	8.83	4.18	p < 0.001	
Computer Science	Operating Systems	9.52	8.82	3.82	p < 0.001	
Computer Science	Intro to Computer Science	9.11	7.99	3.61	p < 0.001	
Computer Science	Data Structures	7.39	7.44	-0.1	0.938	✓
Computer Science	Natural Language Processing	7.1	5.26	3.58	p < 0.001	
Computer Science	Discrete Mathematics	8.46	4.17	9.12	p < 0.001	
Computer Science	Algorithms	8.21	3.86	10.4	p < 0.001	
Computer Science	Computer Networks	8.26	2.35	14.51	p < 0.001	
Mathematics	Multivariable Calculus with applications to Economics	8.78	6.53	6.85	p < 0.001	
Mathematics	Mathematics for Statistics and Calculus	9.12	5.43	11.01	p < 0.001	
Mathematics	Calculus with applications to Science and Engineering	7.09	4.3	5.56	p < 0.001	
Psychology	Biopsychology	5.99	8.46	-7.83	p < 0.001	✓
Psychology	Intro to Psychology	8.62	7.48	3.21	0.002	
Psychology	Social Psychology	8.28	6.05	6.44	p < 0.001	
Economics	Corporate Finance	8.43	6.98	4.0	p < 0.001	
Economics	Markets	7.3	6.17	1.99	0.044	
SRPP	Intro to Public Policy	4.39	9.56	-11.14	p < 0.001	✓
SRPP	Applied Data Science using Python	9.79	8.43	4.48	p < 0.001	
SRPP	Human Centered Data Science	9.11	8.17	2.85	0.005	
SRPP	Education and Society	9.25	7.85	12.08	p < 0.001	
SRPP	Intro to the Study of Society	7.82	6.93	2.08	0.043	
SRPP	The Sociology of Entrepreneurship	7.78	6.64	2.3	0.029	

Engineering	Quantitative Synthetic Biology	8.67	8.97	-0.76	0.447	✓
Engineering	Cyberwarfare	6.7	7.83	-3.96	p < 0.001	✓
Engineering	Object Oriented Programming	6.34	7.62	-2.77	0.006	✓
Engineering	Structure and Properties of Civil Engineering Materials	7.12	7.29	-0.35	0.711	✓
Engineering	Project Management	8.09	6.13	4.28	p < 0.001	
Engineering	Modeling and Analysis of Dynamical Systems	9.58	5.49	10.22	p < 0.001	
Political Science	Climate/Change	7.06	8.09	-3.96	p < 0.001	✓
Political Science	Data Analysis	8.93	7.04	3.64	p < 0.001	
Political Science	Political Economy of Institutions	6.5	1.87	9.79	p < 0.001	
Business Organizations and Society	Management and Organizations	7.74	9.42	-5.23	p < 0.001	✓

Supplementary Table 3: **Comparing ChatGPT's average standardized grades to those of university students in 32 different course.** Grades are standardized for each grader of each course to account for grader-specific effects. p-values are calculated using bootstrapped Welch's t-test. Ticks highlight courses where the average grade of GPT submissions is not significantly lower than that of students. SRPP stands for Social Research and Public Policy.

Discipline	Course	Avg. student grade	Avg. GPT grade	t-statistics	p-values	GPT $\geq$ Human
Computer Science	Data Structures	-0.01	0.01	-0.1	0.924	✓
Computer Science	Computer Networks	0.74	-0.74	15.17	p < 0.001	
Computer Science	Algorithms	0.62	-0.62	10.76	p < 0.001	
Computer Science	Discrete Mathematics	0.56	-0.56	9.09	p < 0.001	
Computer Science	Computer Systems Organization	0.3	-0.3	4.19	p < 0.001	
Computer Science	Intro to Computer Science	0.27	-0.27	3.75	p < 0.001	
Computer Science	Natural Language Processing	0.27	-0.27	3.74	p < 0.001	
Computer Science	Operating Systems	0.27	-0.27	3.78	p < 0.001	
Mathematics	Mathematics for Statistics and Calculus	0.63	-0.63	10.95	p < 0.001	
Mathematics	Multivariable Calculus with applications to Economics	0.46	-0.46	6.89	p < 0.001	
Mathematics	Calculus with applications to Science and Engineering	0.38	-0.38	5.57	p < 0.001	
Psychology	Biopsychology	-0.51	0.51	-8.07	p < 0.001	✓
Psychology	Social Psychology	0.44	-0.44	6.52	p < 0.001	
Psychology	Intro to Psychology	0.23	-0.23	3.16	0.002	
Economics	Corporate Finance	0.3	-0.29	4.15	p < 0.001	
Economics	Markets	0.15	-0.15	2.0	0.046	
SRPP	Intro to Public Policy	-0.64	0.64	-11.3	p < 0.001	✓
SRPP	Education and Society	0.67	-0.67	12.1	p < 0.001	
SRPP	Applied Data Science using Python	0.34	-0.34	4.84	p < 0.001	
SRPP	Human Centered Data Science	0.21	-0.21	2.89	0.005	
SRPP	The Sociology of Entrepreneurship	0.18	-0.18	2.43	0.013	

SRPP	Intro to the Study of Society	0.15	-0.15	2.07	0.042	
Engineering	Cyberwarfare	-0.29	0.29	-4.07	p < 0.001	✓
Engineering	Object Oriented Programming	-0.18	0.18	-2.44	0.015	✓
Engineering	Quantitative Synthetic Biology	-0.06	0.06	-0.75	0.45	✓
Engineering	Structure and Properties of Civil Engineering Materials	-0.02	0.02	-0.33	0.73	✓
Engineering	Modeling and Analysis of Dynamical Systems	0.62	-0.62	10.76	p < 0.001	
Engineering	Project Management	0.32	-0.32	4.47	p < 0.001	
Political Science	Climate/Change	-0.24	0.24	-3.29	0.001	✓
Political Science	Political Economy of Institutions	0.59	-0.59	9.8	p < 0.001	
Political Science	Data Analysis	0.26	-0.26	3.64	p < 0.001	
Business Organizations and Society	Management and Organizations	-0.39	0.37	-5.43	p < 0.001	✓

Supplementary Table 4: **Global student survey demographics.**

	Brazil	India	Japan	UK	USA
**Age**					
**18-29**	0.73	0.9	0.88	0.85	0.32
**30-44**	0.19	0.03	0.05	0.09	0.17
**45-60**	0.03	0.02	0.03	0.03	0.41
**> 60**	0.0	0.0	0.0	0.0	0.09
**Gender**					
**Female**	0.5	0.31	0.67	0.7	0.53
**Male**	0.44	0.65	0.29	0.26	0.44
**I prefer not to answer**	0.01	0.0	0.0	0.0	0.02
**Other**	0.0	0.0	0.01	0.01	0.01
**Level of Study**					
**High school**	0.27	0.1	0.2	0.04	0.11
**2-year college**	0.09	0.19	0.06	0.13	0.22
**4-year college**	0.3	0.33	0.54	0.36	0.33
**Certificate**	0.16	0.05	0.01	0.03	0.04
**Masters**	0.06	0.13	0.08	0.18	0.19
**Other graduate program**	0.04	0.14	0.01	0.15	0.08
**Other**	0.08	0.05	0.09	0.09	0.04
**Type of Student**					
**Part time student**	0.54	0.14	0.22	0.14	0.39
**Full time student**	0.4	0.8	0.73	0.84	0.56
**Other**	0.04	0.0	0.04	0.01	0.03
**I do not attend classes but just take exams**	0.02	0.05	0.0	0.01	0.02
**Field of Study (Multiple may apply)**					
**Art**	0.18	0.16	0.06	0.14	0.2
**Humanities**	0.16	0.09	0.15	0.11	0.23
**Social Science**	0.12	0.11	0.14	0.25	0.29
**Natural Science**	0.15	0.13	0.04	0.11	0.18
**Formal Science**	0.18	0.28	0.08	0.11	0.3
**Applied Science**	0.3	0.34	0.23	0.27	0.28
**Other**	0.15	0.11	0.3	0.12	0.1
**Fathers Education**					
**No formal schooling**	0.06	0.03	0.0	0.04	0.06
**Primary School**	0.14	0.03	0.0	0.01	0.04
**Middle School**	0.1	0.07	0.04	0.01	0.03
**Secondary**	0.0	0.25	0.35	0.29	0.2
**High school**	0.26	0.0	0.0	0.0	0.0
**Certificate**	0.17	0.03	0.03	0.12	0.05

**College Degree**	0.14	0.37	0.35	0.35	0.42
**Graduate Degree**	0.07	0.16	0.1	0.14	0.14
**Other**	0.02	0.02	0.1	0.01	0.05
**Self reported socio-economic class**					
**Working class**	0.46	0.05	0.34	0.4	0.31
**Middle class**	0.39	0.79	0.38	0.46	0.51
**Upper class**	0.03	0.07	0.04	0.06	0.1
**Poor**	0.07	0.04	0.07	0.04	0.06
**None of the above**	0.01	0.01	0.14	0.01	0.02

Supplementary Table 5: **Global educator survey demographics.**

	Brazil	India	Japan	UK	USA
**Age**					
**18-29**	0.09	0.43	0.09	0.18	0.21
**30-44**	0.45	0.37	0.28	0.46	0.41
**45-60**	0.39	0.09	0.42	0.24	0.2
**> 60**	0.0	0.01	0.11	0.1	0.15
**Gender**					
**Female**	0.66	0.59	0.41	0.83	0.68
**Male**	0.29	0.31	0.48	0.12	0.27
**I prefer not to answer**	0.0	0.0	0.0	0.01	0.04
**Other**	0.0	0.0	0.0	0.02	0.01
**Education**					
**No formal schooling**	0.0	0.0	0.01	0.0	0.01
**Primary School**	0.01	0.0	0.03	0.01	0.03
**Middle School**	0.0	0.0	0.01	0.01	0.03
**Secondary**	0.0	0.04	0.03	0.1	0.05
**High school**	0.03	0.0	0.0	0.0	0.0
**Certificate**	0.01	0.03	0.05	0.12	0.06
**College Degree**	0.37	0.31	0.54	0.44	0.34
**Graduate Degree**	0.53	0.53	0.21	0.31	0.46
**Other**	0.0	0.0	0.02	0.0	0.02
**Years of experience**					
**< 3 years**	0.09	0.42	0.09	0.16	0.09
**3-5 years**	0.14	0.25	0.07	0.16	0.2
**6-10 years**	0.21	0.21	0.12	0.26	0.29
**11-20 years**	0.33	0.11	0.23	0.26	0.19
**> 20 years**	0.22	0.01	0.47	0.17	0.24
**Level of teaching**					
**Preschool**	0.36	0.06	0.05	0.17	0.19
**1-8 grade students**	0.49	0.38	0.35	0.44	0.54
**9-12 grade students**	0.47	0.37	0.29	0.38	0.3
**Undergraduate students**	0.2	0.28	0.21	0.11	0.25
**Graduate students**	0.07	0.11	0.1	0.07	0.08
**Adults (e.g. (re)training programs/certificates/etc.)**	0.17	0.03	0.07	0.06	0.07
**Other**	0.1	0.0	0.01	0.02	0.01
**Do you grade assignments/homework?**					
**Yes**	0.93	0.9	0.77	0.89	0.92
**No**	0.05	0.02	0.16	0.1	0.07

Supplementary Table 6: **NYUAD student survey demographics.**

	Percentage
**Division**	
**Arts and Humanities**	0.12
**Science**	0.43
**Social Science**	0.24
**Engineering**	0.28
**Study Away Student**	0.03
**Undecided**	0.01
**Gender**	
**Female**	0.41
**Male**	0.56
**Prefer not to say**	0.03
**Other**	0.01
**GPA**	
**2.6-3.0**	0.07
**3.1-3.5**	0.25
**More than 3.6**	0.63
**I prefer not to say**	0.05
**Self reported socioeconomic status**	
**Poor**	0.05
**Working class**	0.15
**Middle class**	0.66
**Upper class**	0.11
**I prefer not to say**	0.03
**Year of study**	
**First**	0.11
**Second**	0.31
**Third**	0.33
**Fourth**	0.19
**"Super Senior"**	0.05

Supplementary Table 7: **NYUAD educator survey demographics.**

	Percentage
**Division**	
**Arts and Humanities**	0.38
**Science**	0.33
**Social Science**	0.25
**Engineering**	0.07
**Type of appointment**	
**Contract**	0.28
**Tenure track without tenure**	0.13
**Visiting**	0.18
**Tenure track with tenure**	0.37
**Other**	0.03
**Length of teaching experience**	
**Less than 3 years**	0.12
**3-5 years**	0.1
**6-10 years**	0.3
**11-20 years**	0.32
**More than 20 years**	0.17
**Gender**	
**Female**	0.4
**Male**	0.52
**Prefer not to say**	0.07
**Other**	0.02

Supplementary Note 1. Survey instruments

Supplementary Note 1.1. Global student survey questions

1. Welcome to this study conducted by Professor Yasir Zaki, Professor Talal Rahwan, and Professor Kinga Makovi and other faculty members at NYU Abu Dhabi!

   The purpose of this brief questionnaire is to learn about how students use technology. The survey will take approximately 5 minutes of your time. There are no correct or incorrect answers, please answer the questions as you see fit.

   We do not collect personally identifying information as part of the study, such as your name or email. Please only participate in the study once.

   There are no harms or benefits in taking part in this study other than those of everyday life.

   If you have questions about the study or your participation, you may contact Kinga Makovi at km2537@nyu.edu. For questions about your rights as a research participant, you may contact the Institutional Review Board (IRB) and refer to HRPP-2023-5, New York University Abu Dhabi, +971 2 628 4313 or IRB-nyuad@nyu.edu.

   Please click on "yes" if you agree to participate in the study and you are 18 years of age or older.

   Do you agree to participate in the study?

   Yes

   No

2. Are you currently a student (i.e., enrolled and taking classes or on leave of absence)?

   Yes

   No

3. Where do you currently study?

   High school

   Certificate training after high school

   2-year college

   4-year college

   Masters program

   Other graduate program e.g., PhD, MBA, JD etc.

   Other

4. Which of the following describes you best?
   - Full time student
   - Part time student
   - I do not attend classes, just take exams
   - Other
5. Which of the following categories best describes your field of study? Please select all that apply.
   - [ ] Arts
   - [ ] Humanities
   - [ ] Social Sciences
   - [ ] Natural Sciences
   - [ ] Formal Sciences (e.g., Computer Science, Mathematics)
   - [ ] Applied Sciences (e.g., Law, Engineering, Medicine, etc.)
   - [ ] Other
6. Compared to other students in your field of study and in your cohort at your institution, where do you fall in terms of academic performance?
   - 5% or less perform better than me
   - 6-10% perform better than me
   - 11-20% perform better than me
   - 21-30% perform better than me
   - 31-40% perform better than me
   - 41-50% perform better than me
   - 51-70% perform better than me
   - >70% perform better than me
7. Do you use the internet in your studies?
   - Yes, often
   - Yes, sometimes
   - No
8. Have you heard of ChatGPT?
   - Yes
   - No

9. Have you ever used ChatGPT for any purpose?

   Yes

   No

10. Have you used ChatGPT to assist with your studies (e.g., homework/assignment/problem set, etc.)?

    Yes

    No

11. Over the past month, did you talk about ChatGPT with your fellow students?

    Yes

    No

12. ChatGPT is a computer program that uses artificial intelligence to understand and respond to text-based prompts and questions. This means it can have a conversation with a person, and provide responses and solutions. Examples of how ChatGPT can be used include:

    Generate text: ChatGPT can generate text that sounds like it was written by a person, such as stories, articles, and even poetry.

    Translate: ChatGPT can translate text from one language to another. Answer questions: ChatGPT can understand and respond to questions, providing information and explanations on a wide variety of topics including history, science, math, etc.

    Summarize text: ChatGPT can read through a document or article and give a brief summary of the main points.

    Code: ChatGPT can write code on different languages such as Python, Java and C++

    ChatGPT has been released recently, and is freely available today for anyone to use, as long as they have an internet connection. Please select "Yes" if you have read the above paragraph.

    Yes

13. Considering your next term of studies, in your field of study, at your institution what share of your peers do you think will use ChatGPT to assist with their studies.

    □

14. Considering your next term of studies, would you use ChatGPT to assist with your studies?

    Yes

    No

15. What is the main reason that you would not use ChatGPT in the next term of your studies?

    I do not know how to use it

    I do not have access to it

    It is unethical to use it

    I am afraid that I will be penalized for using it

    I have no need for it

    Other

16. What is the main reason that you would use ChatGPT in the next term of your studies?

    It will improve my skills (e.g, English writing, coding, etc.)

    It will save me time

    I will earn better grades

    It is fun to use

    I think tools like ChatGPT are the future of education

    Other

17. Since ChatGPT is a new tool, there are very limited guidelines for its use in education. To what degree do you agree or disagree with the following statements? (On a scale from -2 (Strongly disagree) to 2 (Strongly agree))

    Whenever a student uses ChatGPT in their work, they should acknowledge the use of ChatGPT

    It is the responsibility of teachers and professors to detect if ChatGPT was used in students' work

    It is the responsibility of educational institutions to come up with solutions to detect ChatGPT in students' work

    I believe that my institution will be supportive of the usage of ChatGPT to assist students with their studies

    The use of ChatGPT in students' work can be easily detected

The use of ChatGPT should not be allowed in school work regardless of it being acknowledged by the student

ChatGPT will increase the competitiveness of students who are non-native English speakers

The availability of ChatGPT will only make inequalities worse in education

The use of ChatGPT is unethical in the context of homework

I need to learn how to use ChatGPT effectively to improve my school work

ChatGPT is hyped at the moment only because it is new, but soon the hype will be over

I will need to use ChatGPT to be competitive in my future job

I worry that ChatGPT will take my job in the future

In my future job, I will be able to outsource mundane tasks to ChatGPT, while I focus on substantive and creative work

18. What is your gender?

    Male

    Female

    Other

    Prefer not to say

19. Do you work while studying?

    Yes, for pay

    Yes, but not for pay (e.g., unpaid intern, volunteer, etc.)

    No

20. What is the highest level of education of your father?

    No formal schooling

    Primary School

    Middle School

    Secondary (High School)

    Certificate

    Bachelor's Undergraduate College Degree

    Graduate Degree (Master's, PhD, MBA, JD, etc)

    Other

21. How would you characterize your socioeconomic background if you think of the context of your home country?

    Poor

    Working class

    Middle class

    Upper class

    None of the above

Supplementary Note 1.2. Global educator survey questions

1. Welcome to this study conducted by Yasir Zaki, Talal Rahwan, Kinga Makovi and other faculty members at NYU Abu Dhabi! The purpose of this brief questionnaire is to learn about how students use technology. The survey will take approximately 5 minutes of your time. There are no correct or incorrect answers, please answer the questions as you see fit.

   We do not collect personally identifying information as part of the study, such as your name or email. Please only participate in the study once.

   There are no harms or benefits in taking part in this study other than those of everyday life.

   If you have questions about the study or your participation, you may contact Kinga Makovi at km2537@nyu.edu. For questions about your rights as a research participant, you may contact the Institutional Review Board (IRB) and refer to HRPP-2023-XX, New York University Abu Dhabi, +971 2 628 4313 or IRBnyuad@nyu.edu.

   Please click on "yes" if you agree to participate in the study and you are 18 years of age or older.

   Do you agree to participate in the study?

    Yes

2. Do you currently work as a teacher or a professor?

    Yes

    No

3. In the past 5 years, have you worked as a teacher or a professor?

    Yes

    No

4. How many years of experience do you have as a teacher or a professor?

    Less than 3 years

    3-5 years

    6-10 years

    11-20 years

    More than 20 years

    The following questions will ask you about your current job as a teacher or a professor. If you are not currently working as a teacher or a professor, think of your last employment in that role.

5. In your role, do you provide student grading/assessment/feedback on the basis of written work (e.g., grade assignments, homeworks, problem sets, or exams)?

    Yes

    No

6. Typically which group of students do you interact with? Please check all that apply.

    ☐ Preschool/kindergarten students

    ☐ 1-8 grade students

    ☐ 9-12 grade students

    ☐ Undergraduate students (e.g., associate and BA degrees or other qualifications that can be attained after finishing high school)

    ☐ Graduate students (e.g., MS, MBA, PhD etc.)

    ☐ Adults (e.g., (re)training programs, certificates etc.)

    ☐ Other

7. How would you characterize the socioeconomic background of the students whom you interact with? In the boxes include numbers between 0 and 100 to indicate the % of students falling into each category.

    Poor ☐

    Working class ☐

    Middle class ☐

    Upper class ☐

8. Do you use the internet to do your work?

   Yes, often

   Yes, sometimes

   No

9. Have you heard of ChatGPT?

   Yes

   No

10. Have you ever used ChatGPT for any purpose?

    Yes

    No

11. ChatGPT is a computer program that uses artificial intelligence to understand and respond to text-based prompts and questions. This means it can have a conversation with a person, and provide responses and solutions. Examples of how ChatGPT can be used include:

    Generate text: ChatGPT can generate text that sounds like it was written by a person, such as stories, articles, and even poetry.

    Translate: ChatGPT can translate text from one language to another. Answer questions: ChatGPT can understand and respond to questions, providing information and explanations on a wide variety of topics including history, science, math, etc.

    Summarize text: ChatGPT can read through a document or article and give a brief summary of the main points.

    Code: ChatGPT can write code on different languages such as Python, Java and C++

    ChatGPT has been released recently, and is freely available today for anyone to use, as long as they have an internet connection. Please select "Yes" if you have read the above paragraph.

    Yes

12. In the upcoming term, what proportion of your students do you think will use ChatGPT to assist with their school work (e.g., homework, assignment, problem set etc.) at the institution where you work? If you are not currently working as a teacher or a professor, think of your last employment in that role.

13. Since ChatGPT is a new tool, there are very limited guidelines for its use in education. To what degree do you agree or disagree with the following statements? (On a scale from -2 (Strongly disagree) to 2 (Strongly agree))

    Whenever a student uses ChatGPT in their work, they should acknowledge the use of ChatGPT

    It is the responsibility of teachers and professors to detect if ChatGPT was used in students' work

    It is the responsibility of educational institutions to come up with solutions to detect ChatGPT in students' work

    I believe that my institution will be supportive of the usage of ChatGPT to assist students with their studies

    The use of ChatGPT in students' work can be easily detected

    The use of ChatGPT should not be allowed in school work regardless of it being acknowledged by the student

    ChatGPT will increase the competitiveness of students who are non-native English speakers

    The availability of ChatGPT will only make inequalities worse in education

    The use of ChatGPT is unethical in the context of homework

    My students need to learn how to use ChatGPT effectively to improve their school work

    ChatGPT is hyped at the moment only because it is new, but soon the hype will be over

    I will need to use ChatGPT to be competitive in my job

    I worry that ChatGPT will take my job in the future

    In my job, I will be able to outsource mundane tasks to ChatGPT, while I focus on substantive and creative work

14. What is your gender?

    Male

    Female

    Other

    Prefer not to say

15. What is your highest level of education?

    No formal schooling

Primary School

Middle School

Secondary (High School)

Certificate

Bachelor's Undergraduate College Degree

Graduate Degree (Master's, PhD, MBA, JD, etc)

Other

Supplementary Note 1.3. NYUAD student survey questions

1. Welcome to this study conducted by Professor Yasir Zaki, Professor Talal Rahwan, Professor Kinga Makovi and other faculty members at NYU Abu Dhabi! The purpose of this brief questionnaire is to learn about how students use technology. The survey will take approximately 5 minutes of your time. There are no correct or incorrect answers, please answer the questions as you see fit. We do not collect personally identifying information as part of the study, such as your name, netID or email. Please only participate in the study once. There are no harms or benefits in taking part in this study other than those of everyday life. If you have questions about the study or your participation, you may contact Kinga Makovi at km2537@nyu.edu. For questions about your rights as a research participant, you may contact the Institutional Review Board (IRB) and refer to HRPP-2023-5, New York University Abu Dhabi, +971 2 628 4313 or IRBnyuad@nyu.edu. Please click on "yes" if you agree to participate in the study and you are 18 years of age or older. Do you agree to participate in the study?

   Yes

2. Are you currently a student (i.e., enrolled and taking classes or on leave of absence) at NYUAD?

   Yes, I am an NYUAD student

   Yes, I am currently visiting from NYUNY or NYUSH

   No

3. Have you heard of ChatGPT?

   Yes

   No

4. Have you ever used ChatGPT for any purpose?

   Yes

No

5. Have you used ChatGPT to assist with your school work (e.g., homework/assignment/problem set, etc.)?

    Yes

    No

6. Over the past month, did you talk about ChatGPT with your fellow students?

    Yes

    No

7. ChatGPT is a computer program that uses artificial intelligence to understand and respond to text-based prompts and questions. This means it can have a conversation with a person, and provide responses and solutions. Examples of how ChatGPT can be used include:

    Generate text: ChatGPT can generate text that sounds like it was written by a person, such as stories, articles, and even poetry.

    Translate: ChatGPT can translate text from one language to another. Answer questions: ChatGPT can understand and respond to questions, providing information and explanations on a wide variety of topics including history, science, math, etc.

    Summarize text: ChatGPT can read through a document or article and give a brief summary of the main points.

    Code: ChatGPT can write code on different languages such as Python, Java and C++

    ChatGPT has been released recently, and is freely available today for anyone to use, as long as they have an internet connection. Please select "Yes" if you have read the above paragraph.

    Yes

8. Do you plan on using ChatGPT to assist with your assignments?

    Yes, definitely

    Yes, probably

    No, probably not

    No, definitely not

9. Now, think about your peers at NYUAD. What do you think students answered to this question: "Do you plan on using ChatGPT to assist with your assignments?" Please make your best guess by assigning a number to each of the categories between 0 to 100 to express the % of students in each.

   Yes, definitely ☐

   Yes, probably ☐

   No, probably not ☐

   No, definitely not ☐

10. Do you think one should use ChatGPT to assist with one's assignments?

    Yes, definitely

    Yes, probably

    No, probably not

    No, definitely not

11. Now, think about your peers at NYUAD. What do you think students answered to this question: "Do you think one should use ChatGPT to assist with one's assignments?" Please make your best guess by assigning a number to each of the categories between 0 to 100 to express the % of students in each.

    Yes, definitely ☐

    Yes, probably ☐

    No, probably not ☐

    No, definitely not ☐

12. Earlier, you told us that you would be unlikely to use ChatGPT to assist with your assignments. What is the main reason for your answer?

    I do not know how to use it

    I do not have access to it

    It is unethical to use it

    I am afraid that I will be penalized for using it

    I have no need for it

    Other ☐

13. Earlier, you told us that you would be likely to use ChatGPT to assist with your assignments. What is the main reason for your answer?

    It will improve my skills (e.g., English writing, coding, etc.)

It will save me time

I will earn better grades

I think tools like ChatGPT are the future of education

Other [ ]

14. Since ChatGPT is a new tool, there are very limited guidelines for its use in education. To what degree do you agree or disagree with the following statements? (On a scale from -2 (Strongly disagree) to 2 (Strongly agree))

    Whenever a student uses ChatGPT in their work, they should acknowledge the use of ChatGPT

    It is the responsibility of teachers and professors to detect if ChatGPT was used in students' work

    It is the responsibility of educational institutions to come up with solutions to detect ChatGPT in students' work

    I believe that my institution will be supportive of the usage of ChatGPT to assist students with their studies

    The use of ChatGPT in students' work can be easily detected

    The use of ChatGPT should not be allowed in school work regardless of it being acknowledged by the student

    ChatGPT will increase the competitiveness of students who are non-native English speakers

    The availability of ChatGPT will only make inequalities worse in education

    The use of ChatGPT is unethical in the context of homework

    I need to learn how to use ChatGPT effectively to improve my school work

    ChatGPT is hyped at the moment only because it is new, but soon the hype will be over

    I will need to use ChatGPT to be competitive in my future job

    I worry that ChatGPT will take my job in the future

    In my future job, I will be able to outsource mundane tasks to ChatGPT, while I focus on substantive and creative work

15. Have any of your past professors reached out to you about your exam/problem set answers to use as data in a study?

    Yes

    No

16. Have any of your past professors reached out to you to grade anonymous student assignments?

    Yes

    No

17. Which year are you in?

    First

    Second

    Third

    Fourth

    "Super Senior"

18. What is your current current cumulative GPA?

    Less that 1.5

    1.6 - 2.0

    2.1 - 2.5

    2.6-3.0

    3.1 - 3.5

    More than 3.6

    I prefer not to say

19. Which Division offers your major? You may check more than one if you are double majoring across Division.

    - [ ] Arts and Humanities
    - [ ] Engineering
    - [ ] Science
    - [ ] Social Science
    - [ ] Undecided
    - [ ] Study away student

20. What is your gender?

    Male

    Female

    Other

    Prefer not to say

21. How would you characterize your socioeconomic background?

    Poor

    Working class

    Middle class

    Upper class

    None of the above

22. What is your country of origin?

Supplementary Note 1.4. NYUAD educator survey questions

1. Welcome to this study conducted by Professor Yasir Zaki, Professor Talal Rahwan, Professor Kinga Makovi and other faculty members at NYU Abu Dhabi! The purpose of this brief questionnaire is to learn about how students use technology. The survey will take approximately 5 minutes of your time. There are no correct or incorrect answers, please answer the questions as you see fit. We do not collect personally identifying information as part of the study, such as your name, netID or email. Please only participate in the study once. There are no harms or benefits in taking part in this study other than those of everyday life. If you have questions about the study or your participation, you may contact Kinga Makovi at km2537@nyu.edu. For questions about your rights as a research participant, you may contact the Institutional Review Board (IRB) and refer to HRPP-2023-5, New York University Abu Dhabi, +971 2 628 4313 or IRBnyuad@nyu.edu. Please click on "yes" if you agree to participate in the study and you are 18 years of age or older. Do you agree to participate in the study?

    Yes

2. Are you currently a faculty (i.e., enrolled and taking classes or on leave of absence) at NYUAD?

    Yes, I am an NYUAD faculty

    Yes, I am currently visiting NYUAD

    No

3. Have you heard of ChatGPT?

    Yes

    No

4. Have you ever used ChatGPT for any purpose?

    Yes

No

5. Have you used ChatGPT to assist with your work (e.g., creating teaching material, in research or administrative tasks)?

   Yes

   No

6. Over the past month, did you talk about ChatGPT with your colleagues at NYUAD?

   Yes

   No

7. During the next term, how many students do you think will use ChatGPT at NYUAD to assist them with their assignments?

   0-10%

   11-20%

   21-30%

   31-40%

   41-50%

   51-60%

   61-70%

   71-80%

   81-90%

   91-100%

8. ChatGPT is a computer program that uses artificial intelligence to understand and respond to text-based prompts and questions. This means it can have a conversation with a person, and provide responses and solutions. Examples of how ChatGPT can be used include:

   Generate text: ChatGPT can generate text that sounds like it was written by a person, such as stories, articles, and even poetry.

   Translate: ChatGPT can translate text from one language to another. Answer questions: ChatGPT can understand and respond to questions, providing information and explanations on a wide variety of topics including history, science, math, etc.

   Summarize text: ChatGPT can read through a document or article and give a brief summary of the main points.

Code: ChatGPT can write code on different languages such as Python, Java and C++

ChatGPT has been released recently, and is freely available today for anyone to use, as long as they have an internet connection. Please select "Yes" if you have read the above paragraph.

Yes

9. Do you plan on treating the use of ChatGPT as plagiarism in student assignments?

    Yes, definitely

    Yes, probably

    No, probably not

    No, definitely not

10. Now, think about your colleagues at NYUAD. What do you think your colleagues answered to this question: "Do you plan on treating the use of ChatGPT as plagiarism in student assignments?" Please make your best guess by assigning a number to each of the categories between 0 to 100 to express the % of faculty in each.

    Yes, definitely ☐

    Yes, probably ☐

    No, probably not ☐

    No, definitely not ☐

11. Do you think one should treat the use of ChatGPT as plagiarism in student assignments?

    Yes, definitely

    Yes, probably

    No, probably not

    No, definitely not

12. Now, think about your colleagues at NYUAD. What do you think your colleagues answered to this question: "Do you think one should treat the use of ChatGPT as plagiarism in student assignments?" Please make your best guess by assigning a number to each of the categories between 0 to 100 to express the % of faculty in each.

    Yes, definitely ☐

Yes, probably ☐

No, probably not ☐

No, definitely not ☐

13. Since ChatGPT is a new tool, there are very limited guidelines for its use in education. To what degree do you agree or disagree with the following statements? (On a scale from -2 (Strongly disagree) to 2 (Strongly agree))

    Whenever a student uses ChatGPT in their work, they should acknowledge the use of ChatGPT

    It is the responsibility of teachers and professors to detect if ChatGPT was used in students' work

    It is the responsibility of educational institutions to come up with solutions to detect ChatGPT in students' work

    I believe that my institution will be supportive of the usage of ChatGPT to assist students with their studies

    The use of ChatGPT in students' work can be easily detected

    The use of ChatGPT should not be allowed in school work regardless of it being acknowledged by the student

    ChatGPT will increase the competitiveness of students who are non-native English speakers

    The availability of ChatGPT will only make inequalities worse in education

    The use of ChatGPT is unethical in the context of homework

    My students need to learn how to use ChatGPT effectively to improve their school work

    ChatGPT is hyped at the moment only because it is new, but soon the hype will be over

    I will need to use ChatGPT to be competitive in my job

    I worry that ChatGPT will take my job in the future

    In my job, I will be able to outsource mundane tasks to ChatGPT, while I focus on substantive and creative work

14. Have you submitted or are you planning on submitting graded submissions of student assignments to participate in a research study initiated by Yasir Zaki and Talal Rahwan?

    Yes

    No

15. Which division are you affiliated with? Please check all that apply.

    - [ ] Arts and Humanities
    - [ ] Engineering
    - [ ] Science
    - [ ] Social Science

16. How many years of experience do you have teaching in a faculty capacity (i.e, not counting teaching experience during graduate school, etc.)?

    Less than 3 years

    3-5 years

    6-10 years

    11-20 years

    More than 20 years

17. What is your gender?

    Male

    Female

    Other

    Prefer not to say

18. What best describes your appointment at NYUAD?

    Tenure track without tenure

    Tenure track with tenure

    Contract

    Visiting

    Other

19. What is your country of origin?

20. Which country did you obtain your highest level of educational qualification in?

Supplementary Figures

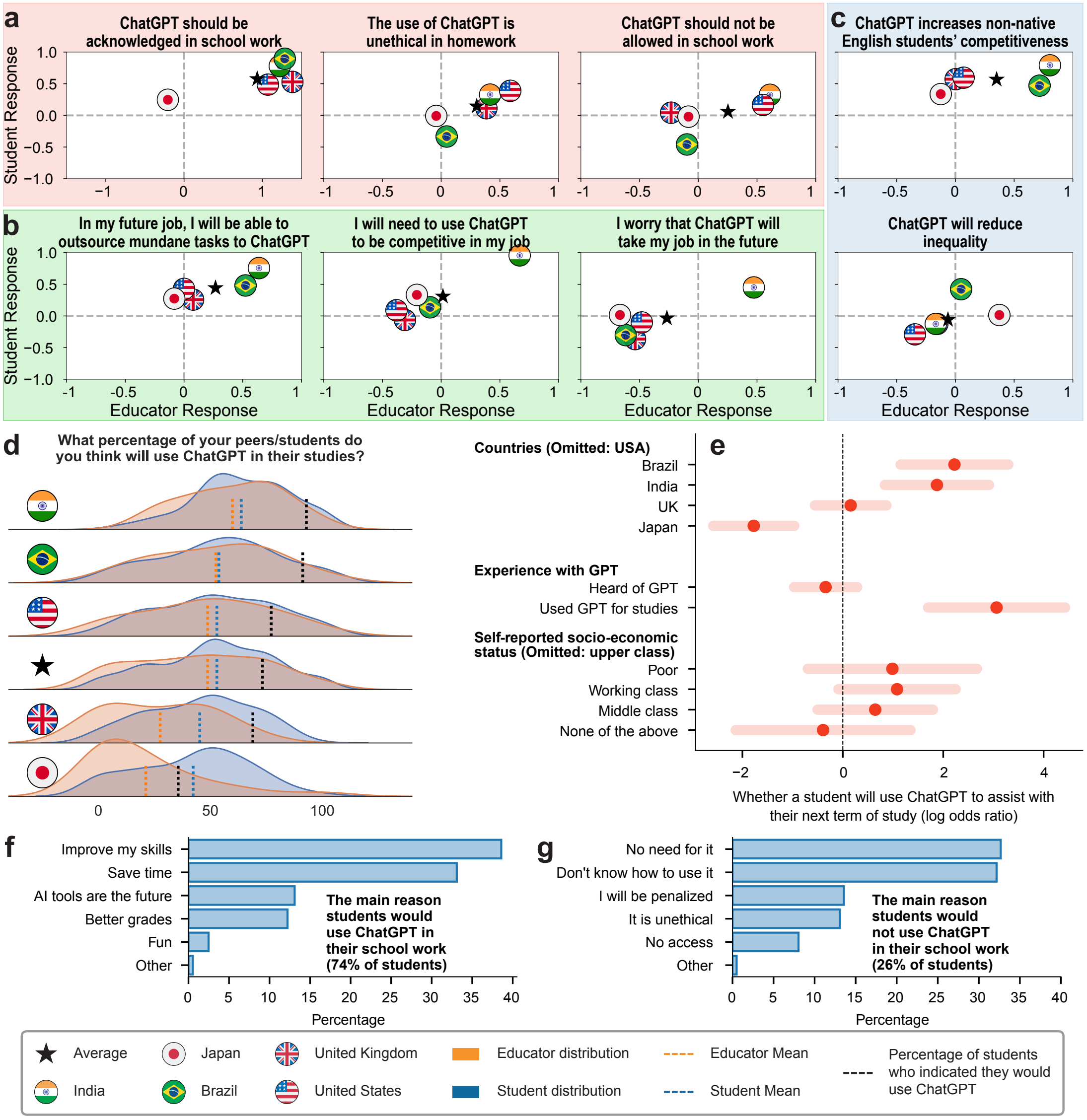

Supplementary Figure 1: **Global survey responses.** (**A-C**) University educators' average responses (x-axis) and university students' average responses (y-axis) to eight questions regarding ChatGPT; $1 = \text{agree}$; $-1 = \text{disagree}$; $\star = $ average over the five countries. (**D**) Distributions of students'/educators' estimation of the percentage of their peers/students who they believe will use ChatGPT in their studies. The dashed black line represents the percentage of students who indicated they would use ChatGPT in their studies. (**E**) OLS-estimated coefficients and 95% confidence intervals of selected independent variables predicting a student's decision to use ChatGPT in their next term of studies; see Supplementary Figure 3 for all independent variables considered. (**F-G**) A breakdown of why students indicated they would or would not use ChatGPT.

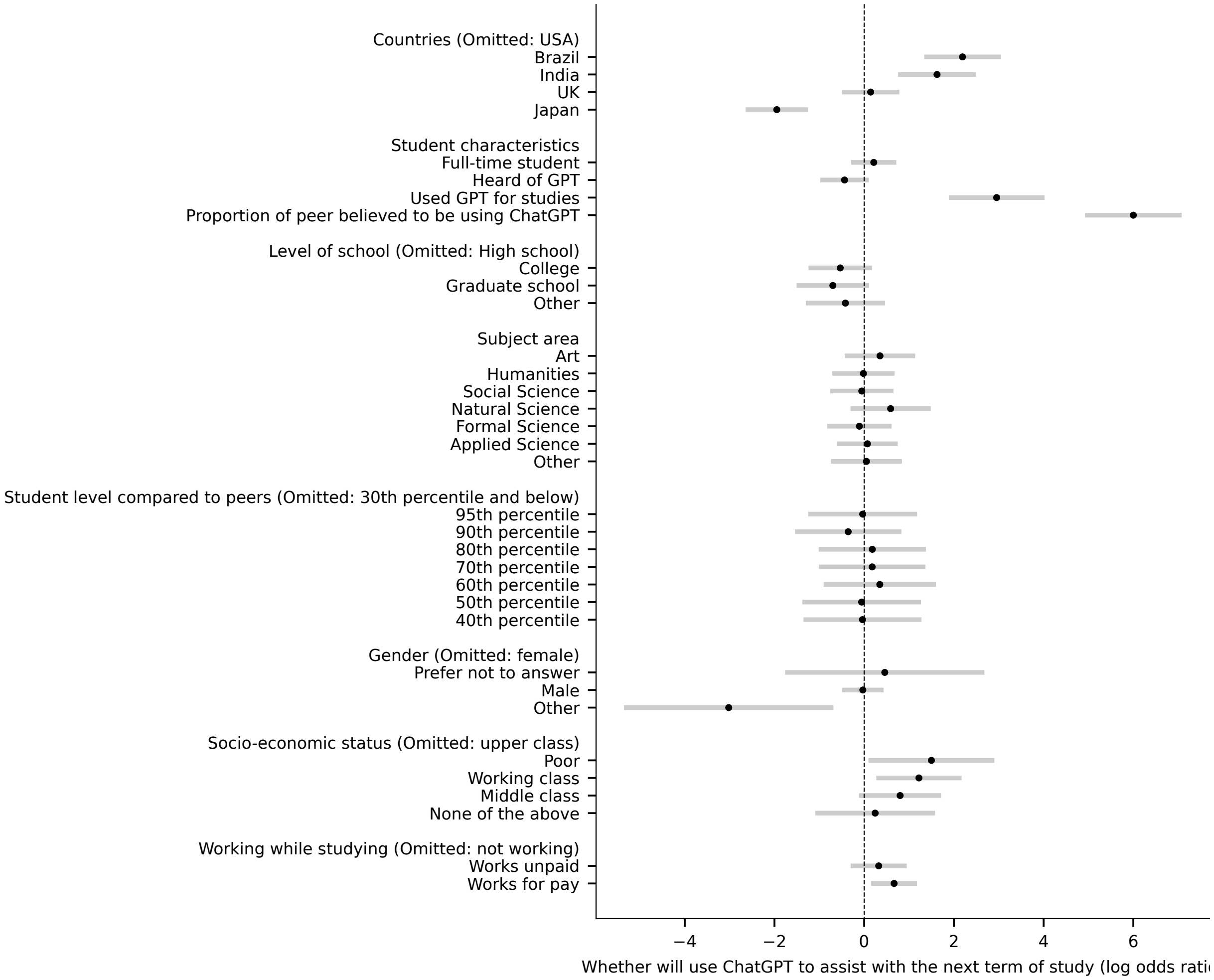

Supplementary Figure 2: OLS-estimated coefficients and 95% confidence intervals of selected independent variables predicting a student's decision to use ChatGPT in their next term of studies.

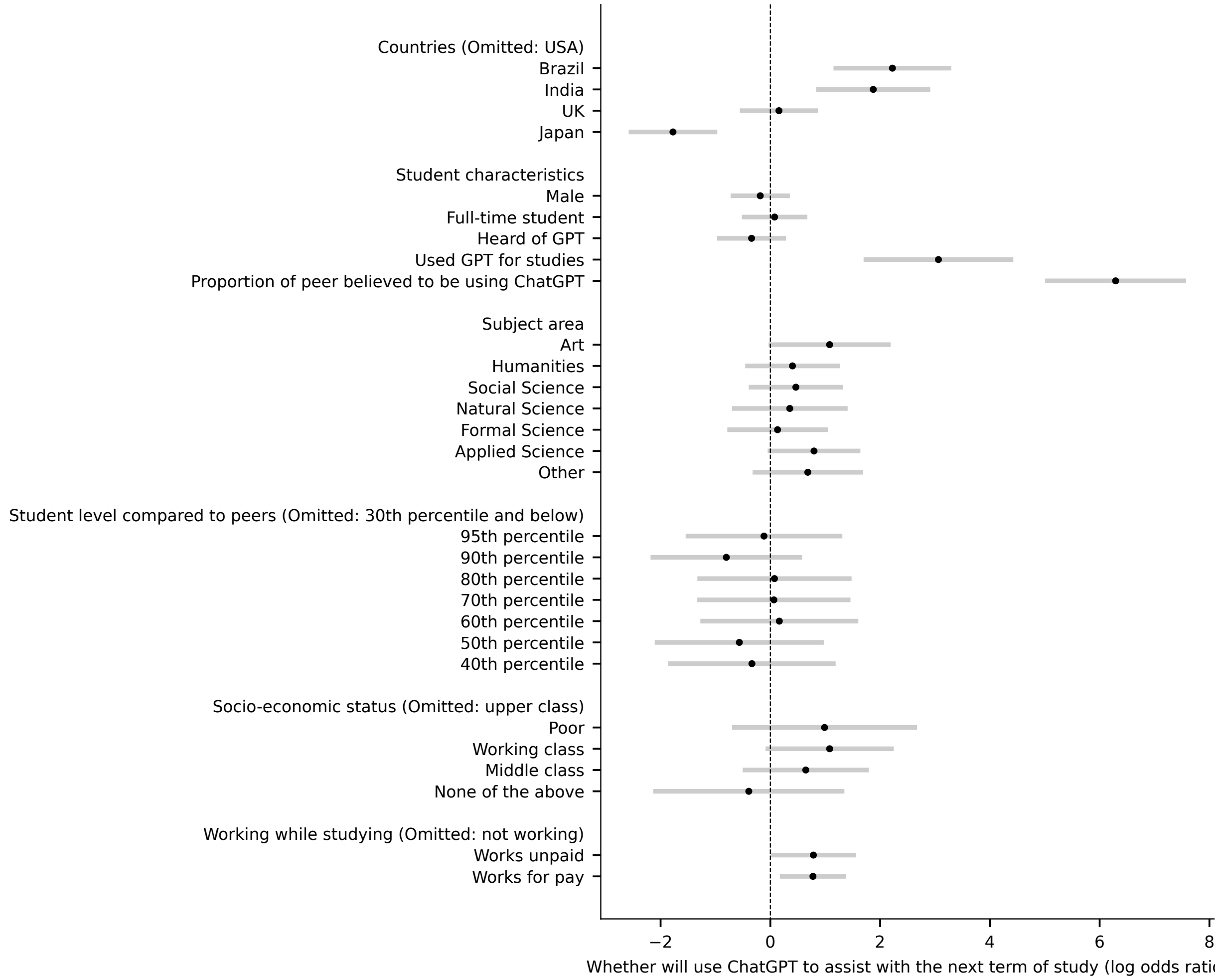

Supplementary Figure 3: OLS-estimated coefficients and 95% confidence intervals of selected independent variables predicting a university or postgraduate student's decision to use ChatGPT in their next term of studies.

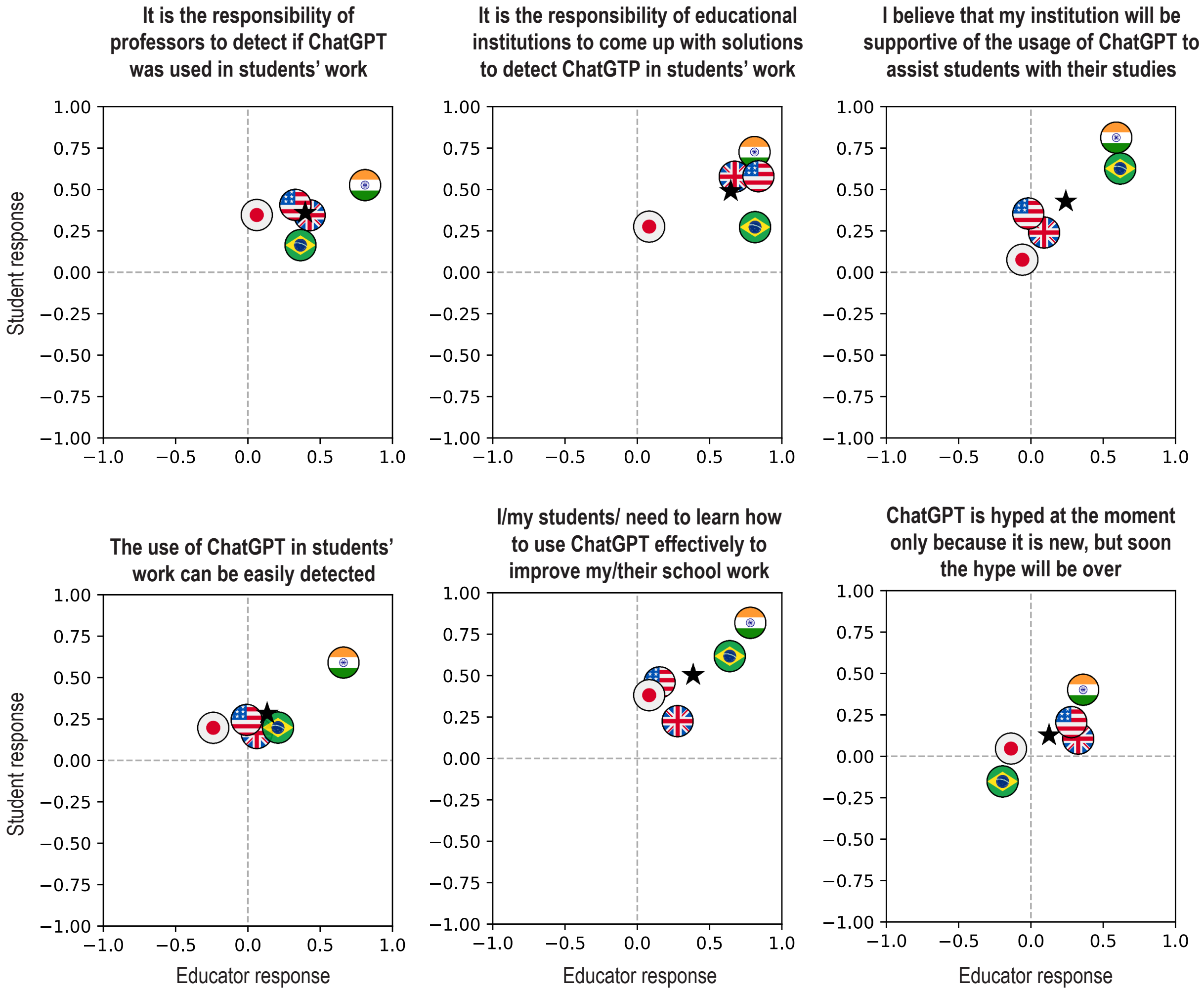

Supplementary Figure 4: Educators' average responses (x-axis) and students' average responses (y-axis) to six remaining questions regarding ChatGPT; 1 = agree; -1 = disagree; star = average over the five countries.

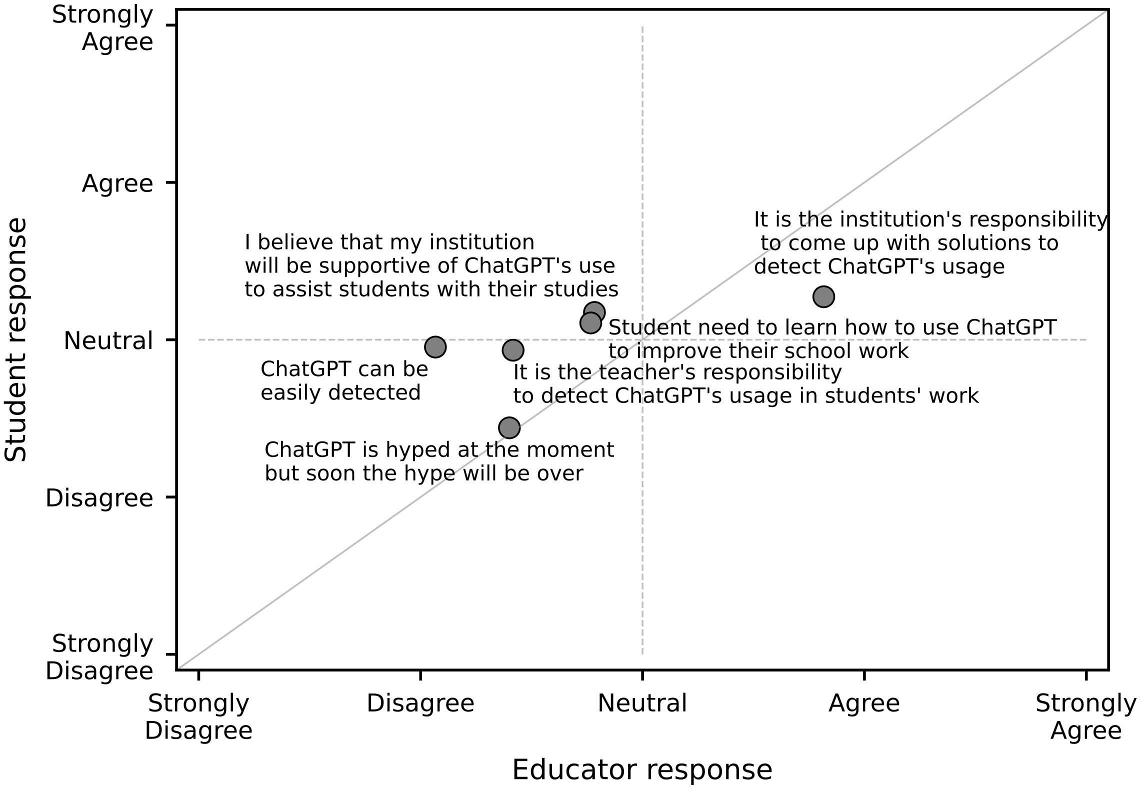

Supplementary Figure 5: Professors' responses (x-axis) and Students' responses (y-axis) to the six remaining statements regarding ChatGPT.

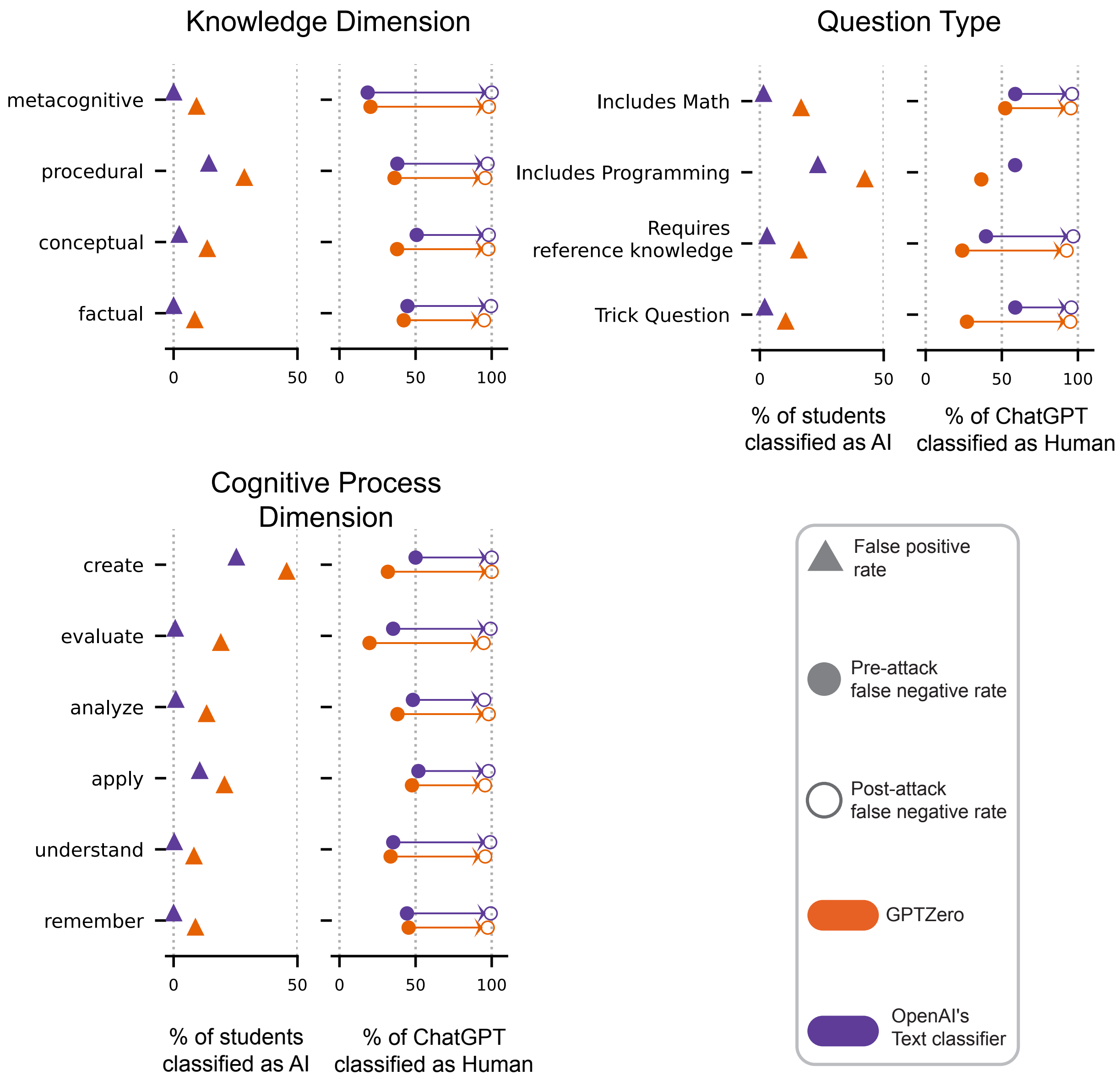

Supplementary Figure 6: Evaluating the two classifiers across the "cognitive process" and "knowledge" dimensions of Anderson and Krathwohl taxonomy's of learning, as well as the type of question.
